# Examining the effect of authentic leadership and contextual performance on work engagement among employees working in sports organizations

**DOI:** 10.3389/fpsyg.2026.1669534

**Published:** 2026-03-25

**Authors:** Uğur İnce, Büşra Özcan, Anıl Siyahtaş, Ramazan Erdoğan

**Affiliations:** 1 School of Physical Education and Sports, Harran University, Şanlıurfa, Türkiye; 2 School of Physical Education and Sports, Siirt University, Siirt, Türkiye; 3 Turkish Football Federation, Istanbul, Türkiye; 4 Faculty of Sports Sciences, Munzur University, Tunceli, Türkiye

**Keywords:** authentic leadership, contextual performance, employee motivation, leadership styles, sports, work engagement

## Abstract

**Background:**

This study examines the relationship between authentic leadership and employees’ contextual performance and investigates its association with work engagement. Specifically, the aim was to explore how authentic leadership practices are associated with employee motivation and job performance in the sports sector.

**Methods:**

The study employed a quantitative research method using measures of authentic leadership, work engagement, and contextual performance. Data were collected through surveys administered to participants, and various statistical analyses were conducted. A mediation analysis was conducted to examine whether work engagement mediates the relationship between authentic leadership and contextual performance.

**Results:**

The analysis showed that authentic leadership is positively associated with work engagement (*β* = 0.516; *p* < 0.001). Work engagement is also positively associated with contextual performance (*β* = 0.393; *p* < 0.001). Mediation analysis suggested that authentic leadership is indirectly associated with contextual performance through work engagement [indirect effect *β* = 0.205; 95% CI (0.152, 0.259); *p* < 0.001], while the direct association between authentic leadership and contextual performance remained significant (*β* = 0.311; *p* < 0.001).

**Conclusion:**

The study’s results indicate that authentic leadership is positively associated with contextual performance via higher employee engagement in the sports sector. The trusting environment and supportive communication provided by authentic leaders are associated with higher employee motivation and linked to better performance outcomes. The findings suggest that sports managers may benefit from cultivating authentic leadership qualities, which are likely to be associated with greater organizational effectiveness.

## 1 Introduction

The complex nature of management in organizations operating in open-system industries, particularly due to the relationship between human resource management and leadership style, has led to the importance of leadership type in sports institutions (Hogan and Kaiser, 2005; Nanjundeswaraswamy and Swamy, 2014). The literature has identified relationships between authentic leadership, work engagement, and contextual performance. Numerous studies have been conducted on the relationships between authentic leadership and work engagement (Penger and Cerne, 2014; Oh et al., 2018), authentic leadership and contextual performance (Malik, 2018), and work engagement and contextual performance (Cesário and Chambel, 2017; Kartal, 2018; Meyers et al., 2020). While previous studies in Türkiye have examined the relationships between authentic leadership, work engagement, and contextual performance, most of these studies have addressed these variables separately or within different organizational contexts (Çetin et al., 2013; Korku C. and Kaya S., 2020; Korku M. and Kaya N., 2020; Korku and Yıldız, 2023). Although limited research has examined the mediating role of work engagement in the association between authentic leadership and contextual performance, theoretical and empirical evidence suggests that leadership behaviors are associated with employee motivation and extra-role performance. According to Avolio and Gardner (2005), authentic leadership fosters trust, transparency, and employee engagement, which are likely to function as mechanisms linked to contextual performance outcomes. Schaufeli et al. (2002) also highlight the centrality of work engagement in positive employee outcomes, suggesting that engaged employees demonstrate higher levels of voluntary and extra-role behaviors. Therefore, this study proposes that work engagement may serve as a mediator in the association between authentic leadership and contextual performance, particularly in public sports organizations where leadership behaviors are associated with employee motivation and organizational effectiveness. This approach provides a deductive, mechanism-based rationale for the proposed hypotheses, rather than relying on the absence of prior studies.

In recent years, authentic leadership has received increasing attention and emerged as an important leadership model (Hsieh and Wang, 2015). Various studies have demonstrated that authentic leadership is positively associated with employee attitudes, behaviors, and business outcomes (Hsieh and Wang, 2015; Ribeiro et al., 2018; Hussein et al., 2016; Iqbal et al., 2015). Authentic leaders encourage employees to engage with their work and exhibit contextual behaviors beyond their official duties. Contextual behaviors are shaped by motivational factors; in this context, engagement stands out as a factor that is associated with higher employee motivation (Meyers et al., 2020). Authentic leaders are associated with higher levels of performance by encouraging employees and the organization to discover and utilize their strengths (Hadian Nasab and Afshari, 2019). Numerous studies have demonstrated the positive relationships of authentic leadership with employee motivation and commitment (Walumbwa et al., 2008). These associations appear to become more pronounced in dynamic and competitive environments such as sports organizations (Korku C. and Kaya S., 2020; Korku M. and Kaya N., 2020). Research shows a positive relationship between authentic leadership and engagement (Penger and Cerne, 2014; Oh et al., 2018). Authentic leaders have the ability to create a supportive environment by recognizing employees’ talents and strengths (Hsieh and Wang, 2015). Recent studies have found that authentic leadership is positively associated with employee engagement levels, particularly in remote work environments (Ribeiro et al., 2021). Contextual behaviors have been shown to be associated with employee motivation, and work engagement is positively associated with contextual performance (Meyers et al., 2020; Schaufeli, 2021). Studies such as Cesário and Chambel (2017) and Kartal (2018) have highlighted a significant relationship between work engagement and contextual performance. Research on the relationship between authentic leadership and contextual performance is quite limited. Malik (2018), while examining the association of authentic leadership with overall job performance, suggested that work engagement may serve as a mediator in this relationship. Furthermore, a recent study revealed that authentic leadership is positively associated with employee attitudes, which are linked to contextual performance through lower job stress and higher job satisfaction (Zhao et al., 2022). This suggests that future research should further investigate the associations between authentic leadership, work engagement, and contextual performance. The purpose of this research is to explore the mediating role of work engagement in the association between authentic leadership and contextual performance.

### 1.1 Authentic leadership

Authentic leadership is a leadership style that allows leaders to understand themselves and their followers and to create a safe and supportive environment. Avolio and Gardner (2005) define authentic leadership as one of the positive leadership styles, emphasizing that one of the key dimensions of this type of leadership is the leader’s genuine and sincere behavior. By helping individuals understand their strengths and weaknesses, authentic leaders are associated with the development of both themselves and their followers (Ilies et al., 2005).

Authentic leadership is associated with higher levels of trust and psychological safety among employees. Penger and Cerne (2014) state that authentic leaders create a safe environment, allowing employees to express themselves, which is positively associated with work engagement. Ciftci and Erkanli (2020) state that employees who feel safe and free from pressure tend to exhibit higher levels of commitment under a supportive leader.

The key components of authentic leadership include self-awareness, transparency in relationships, balanced behavior, and an internalized sense of morality. Self-awareness refers to a leader’s awareness of their own strengths and weaknesses and their understanding of their impact on others (Avolio and Gardner, 2005). Transparency in relationships relates to the leader’s ability to openly share their feelings, values, and information; this is associated with higher credibility (Walumbwa et al., 2008).

Balanced behavior is defined as the leader’s ability to base decisions on an objective basis (Walumbwa et al., 2008). Authentic leaders are associated with objective decision-making. Finally, an internalized sense of morality refers to an individual’s internalized values and consistent mindsets (Walumbwa et al., 2008). These fundamental qualities are associated with better support for employees’ personal and professional development.

The associations of authentic leadership can also be observed at the organizational level. Research shows that authentic leadership is positively associated with employees’ motivation and job satisfaction, ultimately being linked to organizational commitment (Walumbwa et al., 2008; Hsieh and Wang, 2015). This leadership style is associated with creating a positive work environment and higher employee commitment and productivity.

### 1.2 Engagement in work

Work engagement is an important concept that reflects employees’ commitment and motivation to their work (Kahn, 1990). Schaufeli et al. (2002) define work engagement as a positive and satisfying social state associated with work, characterized by high levels of energy and mental flexibility. Bakker and Albrecht (2018) state that work engagement is associated with high dedication and focus. Vigor is expressed through high energy and mental flexibility, while dedication is characterized by feelings of enthusiasm, inspiration, and pride in work. Absorption occurs when an employee is fully focused on their work. This combination is associated with higher performance outcomes (Schaufeli et al., 2002).

Work engagement has been shown to be associated with higher creativity, task performance, organizational citizenship behaviors, and customer satisfaction (Bakker and Albrecht, 2018; Christian et al., 2011; Rich et al., 2010; Albrecht et al., 2015). Cesário and Chambel (2017) state that a high level of engagement is associated with better performance.

In summary, work engagement helps employees perform their jobs more efficiently and creates a more positive atmosphere in the workplace. Therefore, contemporary public and private organizations understand the importance of having engaged employees and are developing strategies to achieve this goal.

*Work Engagement*: An employee’s engagement with their job is defined as the happiness and enthusiasm they experience while performing their job. The concept of work engagement, which is still in its developing stage in the literature, is interpreted as the passion of employees for their work and a deep bond they feel towards the organization they work in (Özer et al., 2015: 262).

### 1.3 Contextual performance

The literature reveals that employee performance is a multidimensional construct. Motowidlo and Van Scotter (1994) argued that employee performance should be evaluated along two distinct dimensions: task performance and contextual performance. While task performance relates to an employee’s performance of their defined duties, contextual performance relates to extra-role behaviors that occur outside of these duties and contribute to the organization (Burney and Kline, 2009). Pradhan and Pradhan (2015) state that contextual behaviors are activities that are not included in the official job description but complement the employee’s task behaviors.

Contextual performance is critical to the functioning of organizations because it refers to activities that are voluntary and do not directly contribute to the employee’s technical skills (Meyers et al., 2020). Such extra-role behaviors include attitudes such as supporting colleagues, establishing positive work relationships, and making extra efforts to complete tasks on time (Pradhan and Patnaik, 2018). Additionally, contextual performance reflects employees’ attitudes and compassion in the workplace.

Today, the workplace is not limited to defined tasks; it is expected that employees’ positive behaviors outside of their formal job roles will increase. This increases the association between contextual performance and organizational success and is associated with employees’ motivation levels (Meyers et al., 2020). Contextual performance includes behaviors such as employees’ adaptation to workplace regulations despite challenging conditions, their willingness to perform additional tasks, and their ability to maintain a positive attitude even when faced with negative situations (Meyers et al., 2020).

The importance of contextual performance varies depending on employees’ sources of motivation. Palenzuela et al. (2019) examined the negative relationship between contextual performance and burnout. This finding highlights the importance of taking measures that are associated with higher employee motivation in the workplace. Managers are encouraged to create environments that are associated with employees’ involvement in work and their positive contributions towards a common goal (Pradhan and Patnaik, 2018).

In conclusion, contextual performance is a behavioral category that is associated with organizational success beyond simply performing employees’ duties. Therefore, it is important for managers to find ways that are associated with higher employees’ contextual performance. By directly serving people and their environment, sports organizations are associated with producing sociologically and psychologically integrated individuals, and therefore societies. All stages of sports organizations are people-centered. In these structures, where people are the focal point, it is believed that services delivered entirely with human emotions, without any material expectations, and aligned with contextual motivations are associated with yielding more positive results.

### 1.4 Authentic leadership, work engagement, and contextual performance

Authentic leadership enables the leader to create positive effects on followers by reflecting their own identity and values (Volio, 2010). Avolio (2010) emphasizes that authentic leaders are characterized by self-awareness, transparency in relationships, balanced behavior, and an internalized moral perspective. This leadership style can increase employee engagement by creating an environment that encourages positive behaviors (Ilies et al., 2005). Penger and Cerne (2014) and Oh et al. (2018) demonstrate a strong relationship between authentic leadership and employee engagement levels.

Work engagement refers to employees’ high commitment and motivation to their work. Schaufeli et al. (2002) define work engagement as the dimensions of vitality, dedication, and absorption. In this context, employees with high energy levels can be more effective in performing their tasks (Bakker and Albrecht, 2018). Furthermore, it has been proven that work engagement affects contextual performance; For employees to exhibit extra-role behaviors, their commitment to their work needs to be increased (Cesário and Chambel, 2017; Kartal, 2018; Meyers et al., 2020).

Contextual performance can be defined as positive behaviors of employees that go beyond their job descriptions, and it is thought that authentic leadership can directly influence these behaviors. Malik (2018) states that authentic leadership is a factor that affects employees’ contextual performance and emphasizes that leaders can increase employees’ extra-role behaviors by developing these characteristics. Particularly in the sports sector, cooperation among employees and the display of extra-role behaviors are critical to the quality of sports services.

High levels of job stress and pressure experienced by employees in sports organizations may negatively affect their overall level of work engagement. In this context, authentic leadership plays a critical role in reducing these negative effects by fostering a sense of psychological safety, trust, and support among employees. By promoting transparency, ethical behavior, and balanced decision-making, authentic leaders can enhance employees’ motivation, engagement, and willingness to display extra-role behaviors.

Although previous studies have examined the relationships between authentic leadership, work engagement, and contextual performance separately, empirical research integrating these variables within a single mediating framework remains limited, particularly in the context of public sports organizations. Existing research has largely focused on private sector employees or general organizational samples, leaving a gap in understanding how leadership behaviors function in performance-oriented and human-centered environments such as sports institutions. Addressing this gap, the present study examines the mediating role of work engagement in the relationship between authentic leadership and contextual performance. By doing so, it contributes to the literature by extending authentic leadership research to the sports sector and by clarifying the psychological mechanisms through which leadership influences employees’ extra-role behaviors and overall organizational effectiveness.

## 2 Material and method

### 2.1 Research pattern

The study employed a quantitative research methodology using scales for authentic leadership, work engagement, and contextual performance. Data were collected through surveys administered to participants, and various statistical analyses were conducted. Furthermore, a mediation analysis was conducted to reveal the relationships between authentic leadership, work engagement, and contextual performance.

### 2.2 Purpose of the research

Sports organizations, like other organizations worldwide, are affected by changing management practices. Leadership is crucial in sports organizations because both the leader and the managed, and the product, are human. The purpose of this research is to examine the mediating role of work engagement in the association between authentic leadership and contextual performance. The primary objectives are:

To examine the association between authentic leadership and work engagement.
To assess the mediating role of work engagement in the association between authentic leadership and contextual performance.
To analyze the relationship between direct and indirect associations of authentic leadership with contextual performance.

### 2.3 Research model

The research model includes a structure that examines the effects of authentic leadership on work engagement and contextual performance. The model includes the following key elements:

Independent Variable: Authentic Leadership.
Mediator Variable: Work Engagement.
Dependent Variable: Contextual Performance.

#### 2.3.1 Hypotheses

The hypotheses to be tested in this study are listed below:

*Hypothesis 1*: Authentic leadership is positively associated with work engagement (*H1*: Authentic Leadership → Work Engagement).

*Hypothesis 2*: Work engagement is positively associated with contextual performance (*H2*: Work Engagement → Contextual Performance).

*Hypothesis 3*: The association between authentic leadership and contextual performance is partially mediated by work engagement (*H3*: Authentic Leadership → Work Engagement → Contextual Performance).

These hypotheses form the basis of the research and provide an important framework for understanding the relationship between authentic leadership and employee contextual performance.

### 2.4 Expected contributions of the research

This study aims not only to reveal the impact of authentic leadership on employee engagement and performance, but also to provide strategic recommendations for managers by providing key insights into positive leadership practices in the sports sector. This will empower sports managers to develop authentic leadership styles and increase employee engagement.

### 2.5 Research group and data collection method

This descriptive study consisted of personnel working at the Istanbul Provincial Directorate of Youth and Sports in 2024. The research population consisted of experts in various professional roles. When the scale forms were examined, any that were left blank or incorrectly completed were eliminated. A total of 361 (158 women and 203 men) participants took part in the study.

### 2.6 Research ethics

The necessary permissions for this research were obtained from the ethics committee of the Siirt University Scientific Research and Publication Ethics Committee with the decision numbered 2023–5,606, dated 04/10/2023. Research permission was obtained from the Ministry of Sports with the permission numbered E-36592570-604.02-5322560, dated 08/03/2023.

### 2.7 Data collection tools

Personal Information Form: An information form was used, which was created by researchers in the light of literature information and included questions such as gender, age, marital status, number of children, length of service at the institution, and level of education of individuals attending sports schools.

#### 2.7.1 Authentic leadership scale

The Authentic Leadership Questionnaire (ALQ), developed by Avolio (2007) and Mind Garden, Inc (2018), was used to assess the authentic leadership characteristics of managers. The scale has been validated as a reliable tool in the Turkish healthcare sector (Korku C. and Kaya S., 2020; Korku M. and Kaya N., 2020). It consists of 16 items across four dimensions: self-awareness, relational transparency, internalized moral perspective, and balanced processing. Items were scored using a frequency-based rating scale ranging from 0 = never to 4 = almost always. Therefore, although sometimes referred to as a “Likert-type” format, the scale is more accurately described as a frequency-based rating scale, consistent with the original instrument.

#### 2.7.2 Work engagement scale

The Utrecht Work Engagement Scale (UWES-17), developed by Schaufeli et al. (2002), was used to measure participants’ work engagement. The Turkish version of the scale has been validated and confirmed as reliable (Eryılmaz and Doğa, 2012). The scale contains 17 items across three dimensions: vigor, dedication, and absorption. Items were scored on a frequency-based rating scale from 0 = never to 6 = almost always. This scoring approach aligns with the original instrument and ensures clarity in interpreting participants’ engagement levels.

#### 2.7.3 Contextual performance scale

The Contextual Performance (CP) subscale of the Employee Performance Scale (EPS), developed by Pradhan and Jena (2017), was used to measure participants’ contextual performance. This unidimensional 10-item scale was scored using a frequency-based rating scale ranging from 0 = never to 4 = almost always. The scale has been validated and its reliability confirmed in the Turkish context (Korku and Yıldız, 2023). All items and scoring procedures were applied according to the original validated source.

Note: All instruments were scored using a frequency-based rating scale, applied consistently with the original validated versions. This clarifies what was measured and how the scores should be interpreted, addressing potential ambiguity in terminology.

### 2.8 Data analysis

The data obtained from the study were analyzed using the Statistical Package for Social Science for Windows (SPSS) 23.0 and Linear Structural Relations (LISREL) 9.30 programs. Cronbach’s Alpha coefficients were examined to determine the reliability of the scales. Descriptive statistics (mean, standard deviation, median, quartile, and percentile) were calculated, and the relationships between the variables were examined using Pearson correlation analysis. Path analysis was conducted to determine the effect of Authentic Leadership (AL) on Contextual Performance (CP) and to examine the mediating role of Work Engagement (WE) in this relationship. The normal distribution of the data was tested by examining kurtosis and skewness values. Kurtosis and skewness for all variables ranged between −1 and +1, and the multivariate kurtosis critical value was below 8, indicating that the assumption of normality was satisfied (Yılmaz and Varol, 2015). In addition to descriptive statistics and correlation analyses, a mediation analysis was conducted to determine whether WE mediates the relationship between AL and CP. The mediation effect was tested using a bootstrapping procedure with 5,000 resamples, as recommended for indirect effect estimation. The results are reported with explicit indirect effect estimates (a × b), 95% bootstrapped confidence intervals (CI), and consistent *p*-values to allow readers to evaluate the mediation claim. Total, direct, and indirect effects were calculated to determine whether the mediation is partial or full, ensuring transparency and interpretability. To assess potential common method variance, Harman’s single-factor test was conducted. The test revealed that the first unrotated factor accounted for 32.4% of the total variance, which is below the 50% threshold commonly used to indicate serious common method bias. Therefore, common method variance does not appear to be a significant concern in this study. Multicollinearity was also examined prior to analysis; variance inflation factor (VIF) values were within acceptable limits, indicating no multicollinearity problem among the study variables. In the present study, the measurement model was tested at the dimension (subscale) level rather than at the individual item level. Specifically, mean scores of each validated sub-dimension were computed and used as observed indicators in the CFA. This parceling approach was preferred to achieve a more parsimonious model structure and to ensure stable parameter estimation given the sample size. All sub-dimensions included in the model correspond to the original validated scale structures. No dimensions were omitted; however, they were represented through aggregated subscale scores rather than individual items. Therefore, CFA results reflect a dimension-level measurement model.

## 3 Results

The mean age of the participants was 35.24 (SD = 6.97), with the age range ranging from 22 to 55. In the gender distribution, men were more represented than women (43.8%) at 56.2%. In terms of marital status, 54.6% of the participants were married, while 45.4% were single. In terms of the number of children, 52.1% of the participants had no children, while 25.8% had one child. When examining the duration of employment at the institution, 21.1% had worked for 1 year or less, 20.5% for 2 to 4 years, and 19.9% for 5 to 7 years. Among university departments, the highest percentage belonged to the Sports Management department at 30.7%. While 78.4% of the participants did not have any postgraduate education, 15.2% had a master’s degree (Table 1).

**Table 1:** Descriptive statistics regarding the demographic characteristics of the participants.

Variables	*n*	%
Age (x̅ ± ss) (Lower-upper value)	35.24 ± 6.97	22–55
Gender		
Female	158	43.8%
Male	203	56.2%
Marital status		
Married	197	56.4%
Single	164	45.4%
Number of children		
None	188	52.1%
1	93	25.8%
2	53	14.7%
3	20	5.5%
4 and above	7	1.9%
Length of service in the institution		
1 year and under	76	21.1%
2–4 year	74	20.5%
5–7 year	72	19.9%
8–10 year	42	11.6%
11–13 year	45	12.5%
14–16 year	18	5.0%
17 years and over	34	9.4%
Department studied at university		
Sports management	111	30.7%
Coaching	54	15.0%
Recreation	24	6.6%
Other	172	47.6%
Postgraduate status		
None	283	78.4%
Master’s Degree	55	15.2%
Master’s Student	21	5.8%
PhD Student	2	0.6%

The mean and standard deviation values of the authentic leadership scale were found to be 3.47 ± 0.75, with the lower and upper values ranging from 1.56 to 5.00, respectively; the mean and standard deviation values of the work engagement scale were found to be 3.70 ± 0.73, with the lower and upper values ranging from 1.65 to 5.00, respectively; the mean and standard deviation values of the contextual performance scale were found to be 4.07 ± 0.53, with the lower and upper values ranging from 2.75 to 5.00, respectively. Cronbach’s Alpha coefficient was interpreted as follows; when 0.00 < *α* < 0.40, the scale was not reliable; when 0.40 < α < 0.60, it had low reliability; when 0.60 < α < 0.80, it was highly reliable; and when 0.80 < α < 1.00, it was highly reliable (Tavakol and Dennick, 2011). When the table was examined, it was seen that the authentic leadership scale and its sub-dimensions, the work engagement scale and its sub-dimensions, and the contextual performance scale had high and high reliability. Tabachnick and Fidell (2013) stated that data were normally distributed when skewness and kurtosis values were between −1.5 and +1.5. When skewness and kurtosis values were examined, it was observed that the authentic leadership scale and its sub-dimensions, the work engagement scale and its sub-dimensions, and the contextual performance scale met the normal distribution assumption (Table 2).

**Table 2:** Statistics of the scales included in the study.

Scales	*x-* ± ss	Lower-upper value	CA (α)	Skewness	Kurtosis
Transparency in relationships (OL1)	3,49 ± 0.84	1.00–5.00	0.866	−0.185	−0.419
Internalized moral understanding (OL2)	3.43 ± 0.79	1.75–5.00	0.787	0.104	−0.405
Balanced assessment/behavior (OL3)	3.43 ± 0.85	1.00–5.00	0.787	−0.139	0.087
Self-awareness (OL4)	3.50 ± 0.79	1.00–5.00	0.829	−0.289	0.214
Authentic leadership scale	**3.47 ± 0.75**	**1.56–5.00**	**0.943**	**0.062**	**0.025**
Desire for work (IA1)	3.76 ± 0.73	1.67–5.00	0.877	−0.353	−0.123
Dedication to work (IA2)	3.69 ± 0.77	1.40–5.00	0.817	−0.345	−0.294
Concentration on work (IA3)	3.65 ± 0.79	1.83–5.00	0.862	−0.009	−0.749
Work engagement scale	**3.70 ± 0.73**	**1.65–5.00**	**0.948**	**−0.169**	**−0.370**
Contextual performance scale	**4.07 ± 0.53**	**2.75–5.00**	**0.925**	**−0.042**	**−0.316**

*x-* ± ss, mean ± standard deviation; CA (α), Cronbach’s Alpha. Bold values indicate the overall scale scores.

Table 3 presents the Pearson correlations among the main study variables, including total scores and sub-dimensions of the Authentic Leadership (AL) and Work Engagement (WE) scales, as well as the Contextual Performance (CP) scale. Sub-dimensions are included for descriptive purposes to provide a more detailed understanding of the relationships. Values close to *r* < 0.20 indicate no or very weak relationship, 0.20–0.39 indicate a weak relationship, 0.40–0.59 indicate a moderate relationship, 0.60–0.79 indicate a high relationship, and 0.80–1.0 indicate a very high relationship (Köklü et al., 2006).

**Table 3:** Analysis of the relationship between the scale and its sub-dimensions used in the study.

		1	2	3	4	5	6	7	8	9	10
1. Transparency in relationships (OL1)	*rh*	1	0.808	0.754	0.736	0.925	0.496	0.418	0.445	0.475	0.501
	*p*		<0.001	<0.001	<0.001	<0.001	<0.001	<0.001	<0.001	<0.001	<0.001
2. Internalized moral understanding (OL2)	*rh*		1	0.706	0.839	0.925	0.513	0.469	0.511	0.523	0.578
	*p*			<0.001	<0.001	<0.001	<0.001	<0.001	<0.001	<0.001	<0.001
3. Balanced assessment/behavior (OL3)	*rh*			1	0.747	0.867	0.419	0.411	0.408	0.431	0.468
	*p*				<0.001	<0.001	<0.001	<0.001	<0.001	<0.001	<0.001
4. Self-awareness (OL4)	*rh*				1	0.909	0.473	0.460	0.457	0.485	0.543
	*p*					<0.001	<0.001	<0.001	<0.001	<0.001	<0.001
5. Authentic leadership scale	*rh*					1	0.527	0.483	0.502	0.528	0.575
	*p*						<0.001	<0.001	<0.001	<0.001	<0.001
6. Desire for work (IA1)	*rh*						1	0.880	0.846	0.950	0.741
	*p*							<0.001	<0.001	<0.001	<0.001
7. Dedication to work (IA2)	*rh*							1	0.885	0.960	0.638
	*p*								<0.001	<0.001	<0.001
8. Concentration on work (IA3)	*rh*								1	0.957	0.611
	*p*									<0.001	<0.001
9. Work engagement scale	*rh*									1	0.694
	*p*										<0.001
10. Contextual performance scale	*rh*										1
	*p*										

Pearson Correlation Test, *p* < 0.05.

A moderately positive relationship was found between AL and WE (*r* = 0.528, *p* < 0.001), and between AL and CP (*r* = 0.575, *p* < 0.001), indicating that as authentic leadership increases, both work engagement and contextual performance also increase. A high positive correlation was observed between WE and CP (*r* = 0.694, *p* < 0.001), suggesting that higher work engagement is associated with higher contextual performance. This table is provided for descriptive and relational purposes only and should not be interpreted as evidence of measurement validity. Discriminant and convergent validity are examined separately using composite reliability (CR), average variance extracted (AVE), and the Fornell-Larcker criterion (see Tables 4, 5).

**Table 4:** Construct reliability and validity.

Factor	CR (composite reliability)	AVE (average variance extracted)
AL	0.82	0.51
WE	0.79	0.52
CP	0.84	0.56

**Table 5:** Discriminant validity (Fornell-Larcker).

Factor	√AVE	AL	WE	CP
AL	0.714	0.714		
WE	0.721	0.541	0.721	
CP	0.748	0.408	0.516	0.748

√AVE values are greater than the correlation between other factors → discriminant validity has been established.

### 3.1 Measurement model specification

To ensure full transparency and eliminate potential ambiguity regarding indicator construction, a detailed mapping between the original validated scales and the indicators used in the CFA is presented in Tables 6, 7.

**Table 6:** Measurement model—CFA loadings, factor covariances, and fit indices.

Factor	Indicator	Estimate (λ)	SE	*Z*	*p*
Authentic leadership (AL)	SA	0.493	0.038	13.1	<0.001
AL	RA	0.581	0.036	16.0	<0.001
AL	IL	0.460	0.026	17.7	<0.001
Work engagement (WE)	VWP	0.612	0.032	19.1	<0.001
WE	DED	0.575	0.035	17.6	<0.001
Contextual performance (CP)	OCB	0.648	0.030	21.6	<0.001
CP	IP	0.604	0.028	20.1	<0.001

AL, Authentic Leadership; WE, Work Engagement; CP, Contextual Performance; SA, Self-Awareness; RA, Relational Authenticity; IL, Internalized Moral Perspective; VWP, Vigor at Work; DED, Dedication; OCB, Organizational Citizenship Behavior, IP, In-Role Performance.

**Table 7:** Mapping of original scales to CFA indicators.

Original scale	Original dimensions/items	Indicator used in CFA	How score was computed	Justification
Authentic Leadership Questionnaire (ALQ)	Self-Awareness (4 items)	SA	Mean of 4 items	Dimension-level CFA to ensure parsimony
	Relational Transparency (4 items)	RT	Mean of 4 items	Original validated structure preserved
	Internalized Moral Perspective (4 items)	IMP	Mean of 4 items	No dimension omitted
	Balanced Processing (4 items)	BP	Mean of 4 items	Aggregation reduces measurement error
Utrecht Work Engagement Scale (UWES-17)	Vigor (6 items)	VIG	Mean of 6 items	Original 3-factor structure retained
	Dedication (5 items)	DED	Mean of 5 items	Dimension-level modeling
	Absorption (6 items)	ABS	Mean of 6 items	Parsimonious representation
Contextual Performance Scale (CP)	10 items (unidimensional)	CP	Mean of 10 items	Modeled as single observed composite

Table 7 explicitly shows:

(i) the original scale structure,
(ii) the number of items per dimension,
(iii) the observed indicators included in the CFA,
(iv) how each indicator score was computed (mean of items), and.
(v) the justification for dimension-level aggregation.

All constructs were modeled at the sub-dimension level in accordance with their original validated structures. No dimensions were omitted. Aggregation was applied to enhance model parsimony and ensure stable parameter estimation relative to the sample size. Accordingly, Authentic Leadership was represented by four indicators (SA, RT, IMP, BP), Work Engagement by three indicators (VIG, DED, ABS), and Contextual Performance as a single composite indicator consistent with its unidimensional 10-item structure.

Confirmatory Factor Analysis (CFA) results, based on the measurement specification described in Table 7, showed that all factor loadings were significant and in the expected direction (see Table 8). Specifically, all standardized loadings exceeded the recommended minimum threshold of 0.40 and were statistically significant (*p* < 0.001), demonstrating sufficient convergent validity at the dimension level. Factor covariances revealed significant positive relationships between Authentic Leadership (AL), Job Engagement (WE), and Contextual Performance (CP), supporting the theoretical consistency of the model. Model fit indices showed excellent fit to the data (*χ*^2^/df = 1.85; CFI = 0.98; TLI = 0.97; RMSEA = 0.045; SRMR = 0.036), all meeting the recommended cutoff criteria (see Table 9). Compound reliability (CR) values exceeded the 0.70 threshold, and Mean Variance Extraction (AVE) values were above 0.50, further supporting construct reliability and convergent validity (see Table 4). Discriminant validity was validated using the Fornell-Larcker criterion, as the square roots of the AVE for each construct were greater than the inter-construct correlations (see Table 5). Overall, these results provide strong evidence that the measurement model is psychometrically robust and suitable for testing the construct mediation model.

**Table 8:** Factor covariances.

Factor 1	Factor 2	Estimate	SE	*Z*	*p*
AL	WE	0.541	0.045	12.02	<0.001
AL	CP	0.408	0.038	10.74	<0.001
WE	CP	0.516	0.043	11.78	<0.001

AL, Authentic Leadership; WE, Work Engagement; CP, Contextual Performance.

**Table 9:** Model fit indices.

Fit index	Value	Recommended cut-off
χ^2^ / df	1.85	<3 good. <5 acceptable
CFI	0.98	≥0.90 good. ≥0.95 very good
TLI	0.97	≥0.90 good. ≥0.95 very good
RMSEA	0.045	<0.08 acceptable. <0.05 very good
SRMR	0.036	<0.08 good

Confirmatory Factor Analysis (CFA) was conducted to test the measurement model for Authentic Leadership (AL), Work Engagement (WE), and Contextual Performance (CP). The factor loadings of all indicators were significant and in the expected direction (SA = 0.493, RA = 0.581, IL = 0.460; VWP = 0.612, DED = 0.575; OCB = 0.648, IP = 0.604; all *p* < 0.001), demonstrating adequate convergent validity. Factor covariances showed significant positive relationships between AL and WE (*r* = 0.541), AL and CP (*r* = 0.408), and WE and CP (*r* = 0.516; all *p* < 0.001), confirming logical consistency among constructs. Model fit indices indicated excellent fit (*χ*^2^/df = 1.85; CFI = 0.98; TLI = 0.97; RMSEA = 0.045; SRMR = 0.036). Construct reliability was satisfactory, with composite reliability (CR) values above 0.70 (AL = 0.82, WE = 0.79, CP = 0.84) and average variance extracted (AVE) values above 0.50 (AL = 0.51, WE = 0.52, CP = 0.56), supporting convergent validity. Discriminant validity was also established, as the square roots of AVE for each construct (AL = 0.714, WE = 0.721, CP = 0.748) were greater than the correlations between constructs. Overall, these results indicate that the measurement model is reliable and valid, providing a robust basis for interpreting the mediation analysis presented in Table 10.

**Table 10:** Examining the mediating effect of work engagement on the effect of authentic leadership on contextual performance.

Model	*R* ^2^	*F*	sd	*β*	SE(β)	*T*	*p*	Bootstrap indirect effect 95% CI	
								95% CI Lower	95% CI Upper
Model 1: ALWE	0.278	138.85	1.359	0.516	0.043	11.783	<0.001	0.429	0.602
Model 2: ALCP (total effect)	0.330	177.50	1.359	0.408	0.030	13.320	<0.001	0.348	0.469
Model 3: AL – WE – CP (direct model)	0.541	211.74	2.358						
AL → CP				0.205	0.029	6.872	<0.001	0.146	0.264
WE → CP				0.393	0.030	12.842	<0.001	0.333	0.453
Indirect effect (AL → WE → CP)								0.152	0.259

AL, Authentic Leadership; WE, Work Engagement; CP, Contextual Performance.

**Figure 1:**
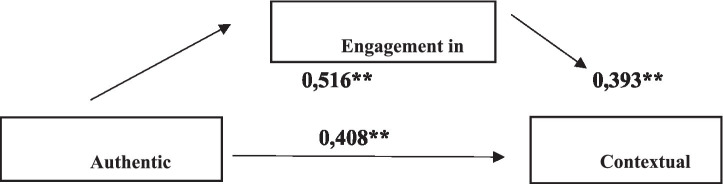
Model of the mediating effect of work engagement on the effect of authentic leadership on contextual performance. ** indicates statistical significance at the 0.01 level (*p* < 0.01).

The results of the mediation analysis examining the effect of Authentic Leadership (AL) on Contextual Performance (CP) through Work Engagement (WE) are presented in Table 10 The effect is considered significant if the 95% bootstrap confidence interval does not include zero. AL significantly predicted WE [*β* = 0.516; *t* = 11.783; *p* < 0.001; 95% CI (0.429, 0.602)] and CP [total effect: *β* = 0.408; *t* = 13.320; *p* < 0.001; 95% CI (0.348, 0.469)]. When WE was included as a mediator, the direct effect of AL on CP decreased but remained significant [*β* = 0.205; *t* = 6.872; *p* < 0.001; 95% CI (0.146, 0.264)], indicating partial mediation. The bootstrap analysis further showed that the indirect effect of AL on CP through WE was statistically significant [*β* = 0.203; 95% CI (0.152, 0.259)], as the confidence interval did not include zero. The reduction of the direct effect from *β* = 0.408 (total effect) to *β* = 0.205 after inclusion of the mediator further supports the presence of partial mediation. Overall, these findings support a partial mediation model, demonstrating that work engagement explains part of the association between authentic leadership and contextual performance, while a significant direct relationship remains (see Figure 1).

This study examines the mediating role of work engagement in the relationship between authentic leadership and contextual performance. The model illustrates the associations among authentic leadership, work engagement, and contextual performance, clearly outlining the role of each variable.

### 3.2 Components of the model

#### 3.2.1 Authentic leadership

Authentic leadership, the independent variable in this model, is defined by the leader’s self-awareness, transparency in relationships, balanced behavior, and internalized moral perspective. Authentic leaders are associated with higher employee motivation by fostering trust and creating a supportive communication environment (Avolio and Gardner, 2005).

#### 3.2.2 Work engagement

Authentic leadership was positively associated with employees’ work engagement (*β* = 0.516; *t* = 11.783; *p* < 0.001), suggesting that supportive leadership behaviors are linked to higher employee commitment to their work.

#### 3.2.3 Contextual performance

Work engagement acted as a mediating variable and was positively associated with contextual performance (*β* = 0.408; *t* = 13.320; *p* < 0.001). Contextual performance includes voluntary behaviors that go beyond employees’ formal job duties, contributing to higher service quality and team cohesion (Meyers et al., 2020).

Mediation results indicate that authentic leadership is directly associated with contextual performance (*β* = 0.205; *t* = 6.872; *p* < 0.001). Additionally, the indirect effect of authentic leadership on contextual performance through work engagement was significant [*β* = 0.203; 95% CI (0.152, 0.259)], demonstrating that work engagement partially mediates this relationship. These results suggest that authentic leadership is associated with contextual performance both directly and indirectly via work engagement.

## 4 Discussion and conclusion

While previous studies in Türkiye have examined the relationships between authentic leadership, work engagement, and contextual performance, most of these studies have addressed these variables separately or within different organizational contexts (Çetin et al., 2013; Korku C. and Kaya S., 2020; Korku M. and Kaya N., 2020; Korku and Yıldız, 2023; Özsarı and Fişekçioğlu, 2020). The present study extends this body of research by integrating these constructs into a single mediation model and empirically testing it within the context of public sports organizations. Unlike earlier research that primarily focused on private sector employees or general organizational samples, this study provides evidence from a human-centered and performance-oriented public sports setting, where leadership behaviors are associated with service quality and employee interaction. The findings reveal that work engagement partially mediates the association between authentic leadership and contextual performance. This result is consistent with previous studies demonstrating that leadership behaviors are associated with employee performance through psychological mechanisms such as work engagement (Saks, 2006; Breevaart et al., 2015; Tims et al., 2011). Accordingly, the present study contributes to the literature by clarifying the explanatory role of work engagement in linking authentic leadership to contextual performance in public sports organizations.

### 4.1 Hypothesis 1: authentic leadership has a positive and significant effect on work engagement

The results of the study reveal that authentic leadership has a strong and significant association with work engagement (*β* = 0.516; *t* = 11.783; *p* < 0.001). The trusting environment and strong communication skills that authentic leaders provide their employees increase their feelings of satisfaction and motivation toward their work. This supports the principles of authentic leadership outlined by Avolio and Gardner (2005), which are associated with higher levels of job satisfaction and motivation among employees. The emphasis on relationships and transparent communication makes employees feel more valued, increasing their commitment to their work. These findings demonstrate the positive association of authentic leadership within organizations, which is particularly meaningful in the context of sports organizations, where uncertainty and career-related concerns have intensified in recent years. Recent evidence suggests that external crises such as the COVID-19 pandemic have substantially reshaped sport management students’ professional self-concepts, career expectations, and perceptions of the work environment (Glebova and López-Carril, 2023), further highlighting the importance of authentic leadership behaviors in fostering trust, engagement, and psychological stability.

### 4.2 Hypothesis 2: work engagement has a positive and significant effect on contextual performance

The study’s findings indicate that work engagement is significantly associated with contextual performance (*β* = 0.408; *t* = 13.320; *p* < 0.001). This suggests that employees’ commitment to their work is linked to extra-role behaviors, such as social interactions and additional duties beyond formal job requirements. High levels of employee engagement are associated with greater individual performance and contribute to overall organizational productivity (Bakker and Albrecht, 2018). Engaged employees tend to go beyond their job descriptions, voluntarily support colleagues, create a positive work atmosphere, and improve work processes, indicating that contextual performance is a key element associated with workforce quality.

### 4.3 Hypothesis 3: the effect of authentic leadership on contextual performance is mediated by work engagement

Analyses reveal that authentic leadership is positively associated with contextual performance, partially through work engagement [direct effect *β* = 0.205; *t* = 6.872; *p* < 0.001; indirect effect *β* = 0.203; 95% CI (0.152, 0.259)]. This finding suggests that the motivational and supportive environment provided by authentic leadership is associated with employees’ commitment to their work. Work engagement is a critical mediator in the association between leadership style and employee performance. Authentic leadership enables employees to express themselves, be emotionally invested in their work, and develop positive social relationships, thereby being associated with higher contextual performance (Malik, 2018). This underscores that developing leaders’ authentic qualities is a critical strategy for organizational success.

In conclusion, this research demonstrates that authentic leadership is positively associated with employees in the sports sector. Higher levels of employees’ motivation are associated with authentic leadership and linked to higher individual performance and stronger organizational commitment. This demonstrates the importance of sports managers developing authentic leadership skills and highlights the role of this leadership style in being associated with enhanced employee job performance.

In management practice, embracing authentic leadership can be associated with higher employee commitment to their work, which is linked to higher organizational productivity. In sports services, the importance of leaders creating a supportive and transparent communication environment is highlighted. This type of environment helps employees reduce stress levels, be more committed to their work, and thus is associated with higher contextual performance.

Future studies need to further explore authentic leadership and employee engagement. In particular, examining the associations of authentic leadership across different sectors and organizational contexts can contribute to identifying universal principles of leadership practices and sectoral differences. Furthermore, the relationship between authentic leadership and employee work-life balance, as well as its association with psychological factors such as burnout and motivation, should also be addressed. This will provide a deeper understanding of the scope of authentic leadership and make its contributions to organizational management practices more evident. The positive association between authentic leadership and employee engagement presents an important strategic approach for managers in the sports sector. Developing and implementing authentic leadership traits can be associated with higher contextual performance through increased employee engagement.

In conclusion, this research demonstrates that authentic leadership is positively associated with employees’ contextual performance both directly and indirectly through work engagement. The findings highlight the importance of authentic leadership behaviors in fostering employee motivation, cooperation, and voluntary performance, particularly in public sports organizations. By strengthening employees’ engagement with their work, authentic leaders are associated with higher organizational effectiveness and service quality.

## 5 Conclusion and recommendations

The findings of this study indicate that authentic leadership is positively associated with employees’ contextual performance, both directly and indirectly through work engagement. Specifically, the results confirm that work engagement serves as a partial mediator in the association between authentic leadership and contextual performance, highlighting the psychological mechanism through which leadership behaviors are associated with extra-role performance. These findings underscore the importance of creating a supportive, transparent, and trust-based leadership environment in sports organizations, where employee motivation and voluntary contribution are essential for organizational effectiveness. By fostering authentic leadership practices, managers can strengthen employees’ engagement with their work, thereby being associated with higher levels of cooperation, responsibility, and performance beyond formal job requirements. Overall, the study contributes to the leadership and organizational behavior literature by demonstrating how authentic leadership operates through work engagement to be associated with higher contextual performance in public sports organizations.

## 6 Limitations and future research

Despite its contributions, this study has several limitations to consider when interpreting its findings. First, the fact that the data were collected from employees of a youth and sports directorate in a single province may limit the generalizability of the results to other regions or organizational contexts. Future studies should replicate the model using samples from different provinces, specific sports organizations, or international settings to increase external validity. The second limitation is that the cross-sectional design of the study restricts the ability to make causal inferences between variables. Longitudinal or experimental research designs may provide stronger evidence about the direction of relationships between authentic leadership, work engagement, and contextual performance. The third limitation is that the data were collected using self-report measures; this may raise concerns about common method bias and social desirability effects. Although statistical controls show that common method variance is not a major issue, future research could benefit from multi-source data such as supervisor evaluations or objective performance indicators. Finally, future studies could expand the model by examining additional mediating or mediating variables such as organizational culture, psychological capital, job stress, or leadership climate. Examining these variables can provide a more comprehensive understanding of how authentic leadership in sports organizations influences employee attitudes and behaviors.

## 7 Recommendations

The findings suggest that leaders, especially in sports organizations, should cultivate authentic qualities. Authentic leadership practices will increase employee job satisfaction and contribute to the creation of a positive workplace atmosphere.

### 7.1 Training and development programs

Authentic leadership training programs are recommended for sports organization managers. These programs should offer strategies for developing leadership skills and enable managers to gain competencies in areas such as empathetic communication, transparency, and building supportive relationships.

### 7.2 Supporting the work environment

Work environments that encourage employee engagement should be created. Particular attention should be paid to stress management and work-life balance, ensuring employees feel secure.

### 7.3 Use of motivational tools

Various reward and recognition systems should be developed to increase employee motivation. These systems should aim to recognize employee efforts and encourage extra-role behaviors.

Future research should examine the relationship between authentic leadership and work engagement in depth across different sectors and cultural contexts. Furthermore, comparing authentic leadership with other leadership styles could make significant contributions to the leadership literature.

## Data Availability

The datasets presented in this study can be found in online repositories. The names of the repository/repositories and accession number(s) can be found in the article/Supplementary material.
