# Supplementary material for: Examining the effect of authentic leadership and contextual performance on work engagement among employees working in sports organizations
Source: Front Psychol. 2026 Mar 25;17:1669534. doi: 10.3389/fpsyg.2026.1669534 (PMC13056639; doi:10.3389/fpsyg.2026.1669534)
Supplement: Supplementary file 1 [file Data_Sheet_1.ZIP › Etik Kurul Kararı (4).pdf]

T.C.
SİİRT ÜNİVERSİTESİ REKTÖRLÜĞÜ

SİİRT ÜNİVERSİTESİ
Bilimin Işığında

BSD4J3ZLYC

ETİK KURULU KARARLARI

Oturum Tarihi	Oturum Saati	Oturum Sayısı
29.09.2023	11:00	665

Üniversitemiz Etik Kurulu 29.09.2023 tarihinde saat 11:00'da Kurul Başkanı Prof. Dr. Cemalettin ERDEMCİ başkanlığında, aşağıda imzaları bulunan üyelerin katılımıyla toplanarak gündemdeki konuları görüşmüş ve aşağıdaki kararları almıştır.

ETİK İNCELEME KONUSU

Dr. Büşra ÖZCAN tarafından yapılacak olan "SPOR KURULUŞLARINDA ÇALIŞAN PERSONELİN OTANTİK LİDERLİK VE BAĞLAMSAL PERFORMANSIN İŞE ANGAJE OLMADA ETKİSİNİN İNCELENMESİ" başlıklı çalışma.

İNCELEME

İnceleme konusu araştırmada, anket yöntemi ile veri toplanarak analizi yapılacaktır. Söz konusu araştırmada, Etik Kurulun görevi kapsamında değerlendirilen husus, araştırma etiğiyle ilgilidir.

Başvurusu yapılan çalışma, insan katılımına dayalı bir araştırmadır. Araştırma etiği bakımından yürütülecek olan programın katılımcıların yararına olması ve onları herhangi bir zarara uğratma riski taşımaması gerekmektedir. Araştırma etiği bakımından ikinci olarak, rıza unsurunun gözetildiğine dair bilgi olmalıdır.

SONUÇ

Sonuç olarak,Sosyal Bilimlerdeki araştırmaların yayın etiği, insan katılımına dayalı olanların da araştırma etiği bakımından etik gereklere uygun olması gerekmektedir. Katılımcıların zarara uğratılmaması temel ilkedir. Araştırmaya katılan ergin bireylerin rızalarının olması halinde toplanan verilerin isim verilmeden raporlaştırılmasında araştırma etiği bakımından bir sakınca bulunmamaktadır.

Kurul Üyeleri:

Prof. Dr. Cemalettin ERDEMCİ
Kurul Başkanı

Doç. Dr. Adnan MEMDUHOĞLU
Kurul Üyesi

Dr. Öğr. Üyesi Arif GÜLLER
Kurul Üyesi

Doç. Dr. Burçak ASLAN ÇELİK
Kurul Üyesi

Dr. Öğr. Üyesi Simla ADAGİDE YILMAZ
Kurul Üyesi

Batman Yolu 10.km Merkez, 56100 Siirt/Türkiye
Tel: +90 (484) 212 11 11
E-Posta: : siu@siirt.edu.tr
Ayrıntılı bilgi için irtibat: Şefik ÇAKIR
Faks: 90 (484) 223 19 98
Elektronik ağ:www.siirt.edu.tr

Bu belge 5070 sayılı Elektronik İmza Kanununun 5. Maddesi gereğince güvenli elektronik imza ile imzalanmıştır.
